# Editorial: Multifunctional Polymeric Materials for Drug and Gene Delivery

**DOI:** 10.3389/fbioe.2022.917690

**Published:** 2022-05-13

**Authors:** Yakai Feng, Bin He, Wenzhong Li, Abraham Jacob Domb

**Affiliations:** ^1^ School of Chemical Engineering and Technology, Tianjin University, Tianjin, China; ^2^ National Engineering Research Center for Biomaterials, Sichuan University, Chengdu, China; ^3^ Institute of Chemistry and Biochemistry, Freie Universität Berlin, Berlin, Germany; ^4^ School of Pharmacy, Faculty of Medicine, Hebrew University of Jerusalem, Jerusalem, Israel

**Keywords:** multifunctional polymer, drug delivery, gene delivery, cancer treatment, targeting

In recent years, various multifunctional polymers have been vastly explored as drug/gene carriers intended to deliver therapeutic reagents (Guo et al., 2019; Duo et al., 2020; Zhang et al., 2020). Their unique physical and biochemical properties render them new functions and possibilities such as targeting, bio-responsiveness, biointeractivity and adaptivity. This Research Topic focuses on recent advances of multifunctional polymers for drug and gene delivery, especially for tissue engineering and regeneration, cancer treatment. There are nine articles, including three review articles and six original research articles. The three review articles summarized the photodynamic therapy, nanogels and polypeptide micelles with tumor microenvironment-responsive drug release, enhanced membrane penetration and gas therapy by generating metabolites of nitric oxide (NO). Wang et al. introduced the smart polymeric delivery systems for the photodynamic therapy of tumor and bacterial infections, mainly discussed the strategies that could be tumor/bacteria targeted or activatable by their microenvironment such as enzyme/pH/reactive oxygen species (ROS). The activation of photodynamic therapy mainly involves following strategies: self-quenching and dequenching of photosensitizers, quenching the triplet state of photosensitizer via using another quencher and dequenching upon cleavage of sensitive bonds, and the responsive change of size and surface charge to enhance the internalization and penetration of tumor cells/bacterial cells. Du et al. summarized tumor microenvironment-responsive nanogels especially their preparation and applications. Nanogels are commonly prepared by free radical polymerization, covalent cross-linking, and physical self-assembly technologies. The drugs can be loaded in nanogels by physical encapsulation and chemical coupling methods. Nanogels endow with unique and useful properties with great potential in chemotherapy owing to their stable size, superior hydrophilicity, excellent biocompatibility, and microenvironment-responsively controlled drug release behaviors (Pinelli et al., 2020). Meanwhile, the authors briefly described the challenges and perspectives of nanogels. Xie et al. introduced the functional polymeric micelles based on basic amino acids i.e., lysine, histidine and arginine, and highlighted their applications as drug carriers for cancer therapy. Polylysine-based polymers with abundant active groups can be used as chemical attachment sites facilitating the construction of drug carriers. Polyhistidine-based polymers having imidazolyl functional groups possess the protonation and deprotonation under different pH-environments, thus enabling pH-responsive drug release. Polyarginine can enhance membrane penetration and gas therapy by generating metabolites of NO (Kudo and Nagasaki, 2015).

The original research articles involved drug/gene delivery for vascular regeneration by sustained release of NO, cancer photothermal and oxidative stress therapy by an NIR-II responsive nanoplatform, nanogel-mediated drug resistance alleviation, ameliorating corneal wound and suppressing neovascularization by Wnt/β-catenin pathway inhibitor and tacrolimus, as well as genetic transformation of plants. These delivery strategies were rationally designed, and demonstrated improvement effects compared with traditional therapeutic methods. Yang et al. developed a nitrate-functionalized vascular graft by electrospinning technology, which could release NO via stepwise biotransformation *in vivo*. This localized delivery of NO demonstrated enhanced cell in growth, endothelial cell monolayer formation, long-termpatency, and vascular smooth muscle cell layer regeneration, whilst inhibiting calcified plaque formation. Thus, the NO release graft could serve as a promising candidate for artificial small-diameter vascular graft replacement and bypass surgery.

ROS can be counteracted by the exorbitant glutathione (GSH) produced by the tumor cells before exerting the antitumor effect of chemodynamic therapy (Liu et al., 2020). Therefore, Huang et al. prepared a thermo-responsive vehicle (NB/CuS@PCM NPs) from borneol (NB) serving as a monoterpenoid sensitizer, and copper sulfide nanoparticles (CuS NPs) as an NIR-II photothermal agent. In the acidic microenvironment, CuS NPs released from vehicle could degrade to Cu^2+^ with the ability of the depletion of GSH. Besides, the copper ion could also convert hydrogen peroxide into hydroxyl radicals for chemodynamic therapy. The combination of oxidative stress–induced damage and photothermal therapy is a potential therapeutic strategy for cancer treatment. To overcome multidrug resistance, Chen et al. developed a mitochondrial-targeting and pH-sensitive nanogel *via* incorporating the hexokinase inhibitor lonidamine and paclitaxel (PTX). The mitochondrial targeting was beneficial for the accumulation and pH-triggered PTX release in the mitochondria. Lonidamine can destroy the mitochondria by exhausting the mitochondrial membrane potential, generating ROS and restraining the energy supply, leading to apoptosis and susceptibility of the cancer cells to PTX. This work provides us a promising and synergistic strategy to conquer tumor multidrug resistance.

Zhong et al. and Lin et al. investigated the Wnt/β-catenin pathway inhibitor XAV939-loaded liposome and tacrolimus-loaded liposome for corneal wound treatment, respectively. These liposomes possessed excellent biological compatibility in human corneal epithelial cells, mouse corneas and eyeballs. The XAV939-loaded liposome demonstrated the antiangiogenic effect, and significantly suppressed the LPS-induced expressions of pro-inflammatory genes. The *in vivo* results also showed that XAV939-loaded liposome ameliorated alkali-burned corneas with slight corneal opacity, reduced neovascularization, and enhanced recovery. The tacrolimus-loaded liposome enhanced corneal epithelial recovery, inhibited corneal neovascularization, and reduced corneal inflammation.

In summary, the current Research Topic reports the recent significant advances in drug and gene delivery with the help of functional polymers. The designing and developing multifunctional polymers will provide new chances for smart drug delivery towards clinical application. These articles in this Research Topic will be a helpful reference for drug/gene delivery.
